# Paediatric trauma systems and their impact on the health outcomes of severely injured children: protocol for a mixed methods cohort study

**DOI:** 10.1186/s13049-016-0260-1

**Published:** 2016-05-13

**Authors:** Kate Curtis, Amy McCarthy, Rebecca Mitchell, Deborah Black, Kim Foster, Stephen Jan, Brian Burns, Gary Tall, Oran Rigby, Russell Gruen, Belinda Kennedy, Andrew J. A. Holland

**Affiliations:** Sydney Nursing School, The University of Sydney, Sydney, NSW Australia; St George Hospital, Kogarah, NSW Australia; George Institute for Global Health, Sydney, Australia; Wollongong Hospital, Wollongong, NSW Australia; Australian Institute of Health Innovation, Macquarie University, Sydney, Australia; Faculty of Health Sciences, The University of Sydney, Sydney, NSW Australia; Faculty of Health, University of Canberra, Canberra, Australia; NSW Aeromedical and Medical Retrieval Services, NSW Ambulance, Sydney, Australia; Sydney Medical School, The University of Sydney, Sydney, Australia; NSW Institute of Trauma and Injury Management, NSW Ministry of Health, Sydney, Australia; Lee Kong Chian School of Medicine, Nanyang Technological University, Singapore, Singapore; Discipline of Paediatrics and Child Health, The Children’s Hospital at Westmead Clinical School, Sydney Medical School, Sydney, Australia; The University of Sydney and The Children’s Hospital at Westmead Burns Research Institute, Sydney, NSW Australia

**Keywords:** Children, Injury, Trauma system, Trauma centers, Triage, Emergency medical services, Ambulances, Outcomes

## Abstract

**Background:**

Injury is a leading cause of death and disability for children. Regionalised trauma systems have improved outcomes for severely injured adults, however the impact of adult orientated trauma systems on the outcomes of severely injured children remains unclear. The objective of this study is to review the processes of care and describe the impacts of a regionalised trauma system on the outcomes of severely injured children.

**Methods:**

This article describes the design of a mixed methods cohort study evaluating the paediatric trauma system in New South Wales (NSW), the most populous state in Australia. Recommendations and an implementation strategy will be developed for aspects of the paediatric trauma care system that require change.

All injured children (aged <16 years) requiring intensive care, or with an Injury Severity Score (ISS) ≥ 9 treated in NSW, or who died following injury in NSW in the 2015–16 financial year, will be eligible for participation. Injury treatment and processes will be examined via retrospective medical record review. Quality of care will be measured via peer review and staff interviews, utilising a human factors framework. Health service and cost outcomes will be calculated using activity based funding data provided by the Ministry of Health. Health-related quality of life (HRQoL) proxy measures will occur at baseline, 6 and 12 months to measure child HRQoL and functional outcomes.

**Discussion:**

This will be the first comprehensive analysis undertaken in Australia of the processes and systems of care for severe paediatric injury. The collaborative research method will encourage clinician, consumer and clinical networks to lead the clinical reform process and will ultimately enable policy makers and service providers to ensure that children seriously injured in Australia have the best opportunity for survival, improved functional outcome and long-term quality of life.

## Background

Injuries are a leading cause of death and disability for children in Australia [1] and worldwide [2]. Disability from severe injury is estimated to occur tenfold for each fatally injured child [2]. Most research has focused on death as an outcome measure. Focusing solely on mortality does not, however, aid our understanding of how the type of care may influence long-term functional, psychological and quality of life outcomes [3]. Injuries significantly impact quality of life across multiple domains, including physical, emotional and psychosocial health [4].

Regionalised trauma systems have resulted in improved mortality and functional outcomes in adults [5]. Evidence describing the impact of trauma systems on the health outcomes for injured children remains limited. The anatomical, physiological and psychological management of injured children varies significantly compared to adults [6, 7]. Stelfox et al. [8] suggested that deficiencies in care exist for up to 45 % of severely injured children and that between 6 % to 32 % of in-hospital deaths may be preventable. It is unclear whether children with severe injury should be transferred to specific paediatric facilities, bypassing adult trauma facilities, or should receive initial stabilisation at an adult trauma centre [9, 10].

The hospital at which an injured child receives treatment typically has been governed by trauma triage protocols and criteria with high rates of over-triage and under-triage reported internationally [11–14]. In New South Wales (NSW), the study site, the most recent data suggest that less than one-third of severely injured children are initially treated at a Paediatric Trauma Centre (PTC) with a survival benefit between three and six times higher if treatment occurred at a PTC compared to those treated at an Adult Trauma Centre (ATC) [15]. The impacts of prehospital triage and transportation destination decision making on the outcomes of severely injured children also remains unclear [10].

The relative costs of health service delivery associated with Australia’s paediatric trauma system have not been investigated. In NSW, the per child average acute treatment cost is $5772 [16], which doubles when a secondary transfer is required [17]. Secondary transfer is required by 70 % of severely injured children (about 100 per year) [15]. One PTC in NSW found that over 60 % of injured children transferred were initially treated in ATCs within the metropolitan area of Sydney [14].

The objective of this study is to review the processes of care within a regionalised trauma system, including the appropriateness of delivery of care, treatment costs and the functional outcomes of children following major and severe injury. Specifically to:

1. determine existing paediatric care pathways from time of injury to definitive care;
2. examine the appropriateness of the processes and delivery of care;
3. determine acute health service delivery costs and the relative costs of different modes of pre-hospital and inter-hospital patient transport – rotary, fixed wing, and road;
4. evaluate the impact of the care pathway on health outcomes by examining patient health-related quality of life (HRQoL) at 6 and 12 months post-injury;
5. integrate findings to identify and prioritise aspects of the paediatric trauma care system that require change; and
6. develop recommendations and a strategy to implement effective, acceptable, feasible change.

## Methods/Design

### Settings

NSW has the highest population of all states and territories in Australia, at June 2015, there were 1.42 million children under 15 years of age, encompassing an area of around 800,000 km^2^ [18]. The transfer and treatment of the injured child in NSW is governed by the NSW Ministry of Health’s state wide trauma services plan [19]. There are three designated PTCs in NSW [20].

Pre- and inter-hospital trauma care is delivered and governed by the NSW Ambulance via land and aeromedical rotary and fixed retrieval. Transport decisions are governed by the pre-hospital trauma (T1) Protocol which provides a state-wide triage process for the prehospital identification of severely injured patients for the transport to definitive treatment at a major trauma centre within a 60 min time frame. Ambulances are to bypass local hospitals when they have a severely injured patient, in preference for a major trauma centre [13]. The protocol does not mandate transport of severely injured children to a PTC.

### Design

**Fig. 1:**
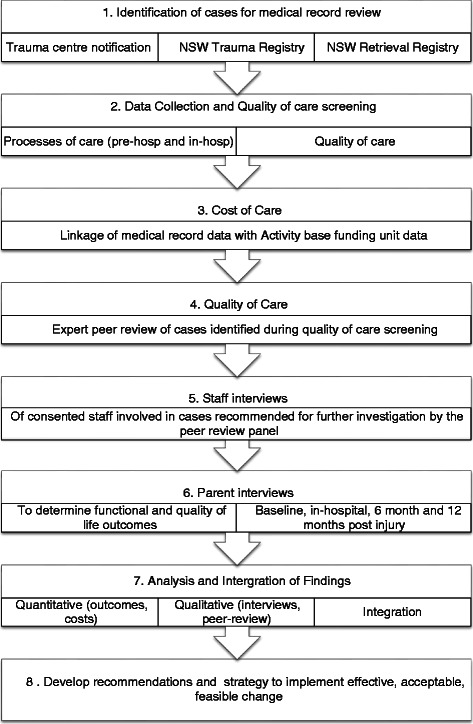
Paediatric trauma system evaluation study process

This mixed methods study will be conducted in four phases, using quantitative and qualitative data. The study process is outlined in Fig. 1.

- Phase 1: To address aims 1, 2 and 3 retrospective reviews of the medical and activity base funding records of all injured children meeting inclusion criteria (see Sampling, recruitment and consent) will occur. This information will map the processes and costs of care for injured children in NSW. Each medical record will be reviewed for appropriateness and quality of care by experienced trauma nurse surveyors (see Data Collection). If any instance of potentially suboptimal care is identified, the case will undergo expert peer review using an evidence-informed, standardised process (see Data Collection).
- Phase 2: The second Phase of the study, also addressing aim 2, will interview consenting staff involved in cases where care is identified by the expert review panel as having been suboptimal. Information from the interviews will be classified using a human factors framework [21] (eg. identifying any issues with equipment, the environment, at an organisational level) and the information from the interviews will be used to identify areas where policy and process change is needed.
- Phase 3: The third Phase of the study is prospective, addresses aim 4, and seeks to determine the 6 and 12 month outcomes of injured children treated at any of the three PTCs in NSW by interviewing their consenting parents regarding their child’s health status and quality of life. The majority of children with major injuries ultimately receive treatment at a PTC.
- Phase 4: Identify and prioritise aspects of the paediatric trauma care system that require change, and develop recommendations and a strategy to implement effective, acceptable, feasible change.

### Sampling, recruitment and consent

There are three groups of participants: child, parent and staff.

### Child participants

All injured children (aged <16 years) requiring intensive care, or with an Injury Severity Score (ISS) ≥ 9 treated in NSW [22], or, who died following injury in NSW in the 2015–16 financial year, will be eligible for participation. In NSW only patients aged < 16 are accepted for treatment in a Paediatric Trauma Centre. Differences of care required among age groups will be reflected in the results, with specific ages grouped and compared. There is no one data source from which to identify severely injured paediatric trauma patients in NSW. Hence, injured participants will be identified through one of four mechanisms outlined in Table 1. A small proportion of children severely injured in rural NSW will likely be transported across borders for treatment due to their proximity to major cities in other states; we will identify these cases using the NSW Medical Retrieval Registry (Air Maestro, Avinet, Australia). Based on 2013 data from all these sources, we anticipate we will review 400 cases. A waiver of consent has been granted to retrospectively review medical records as: (i) obtaining consent from all relevant health consumers for their health information to be included in the study was considered impracticable, and (ii) an incomplete data set would substantially impair the research by introducing bias, and reducing the validity and generalizability of the research to the paediatric trauma population. There will be no contact with the injured children.

**Table 1:** Methods of potential participant identification, date collection and time frame

Method of identification	Trauma centre	Trauma registry	Medical retrieval registry	Coronial data
Time frame post injury	24–48 h	3–6 months	3–6 months	12 months
Medical record review-1^st^ 72 h	ASAP	Delayed	Delayed	Delayed
Medical record review-Full	Post discharge	Delayed	Delayed	Delayed
Baseline-pre-injury estimate	Inpatient-during first two weeks	Not possible	Not possible	N/A
In hospital PedQL and EQ-5D-3 L	Inpatient-during first two weeks	Not possible	Not possible	N/A
6 month PedQL and EQ-5D-3 L	6 months post injury	Not possible	Not possible	N/A
12 month PedQL and EQ-5D-3 L	12 months post injury	Not possible	Not possible	N/A
Staff Interview for peer-review cases	3 months	Not possible	Not possible	Not possible

### Staff participants

Staff involved in the patient phase of care where a potential problem was identified by the expert peer-review process will be invited to participate in a telephone semi-structured interview. The purpose of the interview will be to determine the range of human/clinical and systems based factors that may have contributed to the problem [23]. A general list of potential staff participants (that is, those rostered to the particular work area during the time period of interest) will be provided confidentially by the relevant hospital manager. Written informed consent will be obtained from clinical staff who agree to participate. Of the cases that may require peer-review, there may be up to 6 staff eligible to participate per case.

### Parent participants

Six and 12 month HRQoL of children suffering major injury will be obtained by proxy by interviewing consenting parents. Parents of children with severe injury treated at one of the three PTCs will be eligible for participation. Discussion with the clinical team will include considerations such as the child’s condition and the timing for approaching parents, minimising the risk of any potential coercion. When an appropriate time is identified, the study site trauma coordinator(s) who are assisting with the study will inform parents/caregivers of the study and what their role as participants in the study would entail, including time involved to participate in follow-up telephone questionnaire interviews. Parents will be provided with a participant information package including the participant information sheet, consent form, and the baseline questionnaires (see Data Collection). It will be made clear that participation in the study is voluntary, and the decision not to participate can be made at any time, without reason, and this decision will not affect their child’s care. We anticipate there will be ~ 400 parent groups eligible to participate. A participation rate of approximately 53 % [24] to 86 % [25] is expected (*n* = 200-340).

### Data collection

There are five sources of data collection.

### Phase 1: Injury treatment, processes and costs

#### Medical record review: (data source 1, aim 1)

Trained nurse surveyors will collect information on the processes of care using the child’s medical record. Data will be directly entered into a purpose built secure electronic web-based database that has an accompanying data dictionary. Data include demographics, comorbidities, injury mechanism, ambulance triage details, initial physiology, injuries, and initial and subsequent destinations. The time taken and processes used from injury to key time points will be collected (e.g. time to critical intervention, transport, emergency department (ED) arrival, definitive care). The data will undergo external reliability (test-retest reliability), inter-rater reliability (Cronbach’s alpha) and content validity (peer-review) testing. The template is based on a previous audit tool used by the Evaluation of London Trauma System on Quality and Process of Care [23].

The data collection tool includes defined quality indicators, based on the best available evidence [8]. As an example, a child who has a severely reduced level of consciousness should receive a CT scan of the brain within 1 h. If this did not occur, the trigger will be activated and the case referred for peer-review.

#### Quality of care: peer review: (data source 2, aim 2)

The appropriateness of care will be evaluated by a peer review panel guided by an audit tool consisting of clinical, system and human factor items. The audit tool is based on the World Health Organization (WHO) Guidelines for Trauma Quality Improvement principles [26], the London protocol [23] and human factors classification framework [21, 27]. The London protocol was first published in 1999 and provides evidence-informed instruction to ensure investigation and analysis of an incident beyond the more usual identification of fault and blame. It also aims to use clinical experience and expertise to the fullest extent, and to assist the reflective investigation process [28]. Human Factors contributing to any suboptimal care or events is based on the Human Factors Classification Framework for patient safety developed to allow a systematic approach to analysis of the role of human factors in adverse clinical incidents [21]. The tool and coinciding dictionary will be piloted and validated prior to use.

The peer-review panel is a multidisciplinary clinical team of trauma experts who are independent to the primary research team. Each member of the peer-review team will initially complete the reviews blinded to other panel members, using the validated tool. For any child whose case review does not reach majority consensus including unanimous classification, an interactive session to discuss the case will be conducted with the expert panel until majority consensus (>75 %) on the outcome classification is reached.

#### Health service and cost outcomes (data source 4, aim 3)

A data extract from the Activity Based Funding (ABF) database provided by the NSW Ministry of Health will be used to determine acute patient health care costs [17, 29]. The ABF Taskforce will link and provide length of stay itemised values for all costs associated with in-patient activity, including acute and in-patient rehabilitation, ED and intensive care unit (ICU) episodes. Road ambulance, fixed wing and helicopter costs will be estimated using calculations of annual expenses and clinical staff costs divided by the total missions conducted, total missions per 100,000 population coverage and engine hours. Children transferred from NSW to another state will be identified using the NSW Medical Retrieval Registry with associated costs calculated.

### Phase 2: Quality of care: staff interviews (data source 3, aim 2)

Eligible and consenting staff will participate in a telephone semi-structured interview to determine a range of systems factors such as patient factors, task factors, individual (staff) factors, team factors, work environment factors, organizational and management factors and institutional context factors. Discussions will be audio recorded and include the interviewee’s role in the case; it will be made clear that the purpose of the discussion is not to allocate blame, but is to understand what occurred and to identify any factors that may have contributed to the outcome [28].

### Phase 3: Six and 12 month child functional outcomes: parent interviews (data source 5, aim 4)

Clinical outcomes of children suffering major injury will be obtained by proxy in a structured interview with the child’s parents. Health-related quality of life (HRQoL) data will be collected from recruited via proxy from the parent participants by the nurse surveyors using the Pediatric Quality of Life inventory (PedsQL 4.0) and the EuroQol five-dimensional EQ-5D-3 L™ face-to-face for the pre-injury (baseline) and in-hospital, and by telephone at 6 and 12 months post-injury. Each tool measures different aspects of functioning and well-being post-injury, is validated for use in paediatric injury outcomes research and can be reliably administered via parent proxy, and paper-pencil or telephone [30, 31]. A response rate of between 53 % [24] to 86 % [25] of parents approached is expected.

### Data linkage and management

Data extracts from the NSW Trauma Registry, the NSW Medical Retrieval Registry, coronial information, and patient health care costs will be linked using unique variables, such as medical record number, first and last name, age, gender, date of admission and date of death. This study will use manual record linkage using the available identifiers. Where there is only a partial identifier available, other data variables will also be used in the linkage process. Higher weighting will be given to identifiers, such as first name, last name, date of birth, gender, MRN and hospital during the linkage process.

Each individual in the study will be given a ‘unique study number’ in each data extract, where relevant, and once linked, data will be de-identified. The identifying information (ie, first and last name, MRN and date of birth), along with the unique study number will be kept securely in a password protected file. Further information will only be added to the database should the case require peer-review and/or have the additional 6 and 12 month health outcome data added.

### Data analysis

#### Quantitative data analysis

The primary outcome measure is HRQoL at 12 months post-injury. Secondary outcome measures include mortality, quality of care scores, time to definitive care (arrival at PTC, ICU or time-critical definitive intervention (pre and in-hospital) where indicated and as defined by the expert panel, eg. lifesaving operative intervention, intubation), treatment costs, hospital length of stay (LOS), ICU ventilator days, and complication rates. Mortality and QoL will be risk adjusted using logistic regression and general linear models, as appropriate. Injury severity, clinical stability, geographical location of injury event (eg. rural/urban) will be fixed factors in the analysis. Other covariates of interest for association with 12-month HRQoL include pre-hospital and retrieval response time and level of care, initial hospital level of care, clinical indicators of stability (vital signs, pH, and lactate).

#### Qualitative data analysis

Qualitative data from staff interviews will be managed using NVIVO software [32]. Interviews will be transcribed verbatim. Content analysis methods will be used to analyse the qualitative data which will be organised by phase of care (pre-hospital, retrieval, ED, ICU, operating room, wards), as well as causation. A taxonomy of performance-influencing factors will be developed (based on existing good practice from health and other industries [33]) and applied to ensure consistent and structured consideration of reasons why appropriate care was not delivered. All data will be extracted, coded and categorised and then abstracted into final categories. Mortality and major morbidity will be deemed ‘preventable’, ‘non-preventable’, ‘problems identified (clinical/system/human factors)’, or ‘no problems identified’. The type of hospital (eg trauma centre, rural clinic) and resources available (eg. surgical capability, staffing levels, interventional radiology) will be considered.

#### Data integration

Integration of the quantitative and qualitative components of a mixed methods study is an important aspect of mixed methods research [34]. Connection of each separate set of findings through integration leads to new insights that are not available from individual reporting of phases [35, 36] and will form the basis of the recommendations for the study.

### Phase 4: Development of recommendations and prioritisation for implementation

Based on the results of the data analyses and integration, a series of recommendations to improve pre-hospital transfer, in-hospital service delivery and between-hospital transfers for severely injured children will be developed. To obtain consensus on the suitability and the importance (i.e. prioritisation) of these recommendations, a modified-Delphi study will be conducted with the project’s Translation to Health Care Policy and Practice Group. This group is composed of representatives from each partner organisation, government, NSW ambulance, and consumer groups. The modified-Delphi will consist of two rounds of questionnaires; each questionnaire will be pilot tested on individuals not involved in the research for content ambiguities. In Round 1, the *group* will be invited to rate the suitability and importance of each recommendation (on a 5-point Likert scale), to suggest modifications to recommendations (where relevant), and to list the key factors that led them to rate the recommendations as they did. Determining when a panel of experts has reached consensus is not straightforward, so different levels of consensus will be specified (ie. high, moderate, low) [37]. The panel will be considered to have reached high consensus on a recommendation when the proportion of all of the panel’s ratings reaches ≥70 %, moderate consensus when the proportion is 50–69 %, and low consensus if the proportion is <50 %. In Round 2, the *modified* recommendations will be submitted for rating of suitability and importance [37]. If necessary, a third modified-Delphi round will be conducted.

### Ethics and dissemination

This study protocol was approved by the NSW Population & Health Services Research Ethics Committee; reference number HREC/15/CIPHS/6 and the National Coronial Information Systems via the Department of Justice and Regulation Human Research Ethics Committee; reference number CF/15/18354. Site Specific Approvals were gained at each hospital site.

## Discussion

This will be the first comprehensive integrated mixed-methods study undertaken in Australia of the processes of care and the systems for treating major paediatric injury, the most common cause of death and disability in Australian children. Some Australian paediatric trauma system policies appear outdated [38, 39], inconsistent [40, 41] and, according to findings in NSW, linked to less than ideal outcomes [15]. By evaluating the NSW paediatric trauma system, this study will provide answers to this complex area of health care delivery and generate evidence of international as well as national significance, since the international evidence on the quality of paediatric trauma care delivery is relatively weak. By producing meaningful, practical, acceptable, feasible, sustainable and measurable recommendations, in partnership with influential organisations actively engaged in every step of the paediatric trauma journey, this study will ensure that that the evidence generated is translated into better health policy and practice.

The integration of key human factors concepts into the data collection and analysis, including the role of underlying factors influencing decisions and behaviours, will develop a clearer understanding of what must be addressed to minimise the recurrence of adverse events. The collection and analysis of information about important pre-hospital markers of serious trauma will benefit Ambulance Services in future triage and resource allocation decisions, and will inform the review of national Ambulance Service trauma transport protocols. The peer-review tools and processes developed and validated as part of this project will establish a platform for the ongoing monitoring and continuous improvement of the quality of paediatric trauma care in Australia and in other, similar health systems. The collaborative research method will encourage clinician, consumer and clinical networks to lead the clinical reform process, through shared goals, which will ultimately result in meaningful research and improved outcomes for severely injured children.
